# Systemic Lupus Erythematosus Presenting as Alopecia Areata

**DOI:** 10.7759/cureus.8724

**Published:** 2020-06-20

**Authors:** Parnia Forouzan, Philip R Cohen

**Affiliations:** 1 Dermatology, McGovern Medical School, University of Texas Health Science Center at Houston, Houston, USA; 2 Dermatology, San Diego Family Dermatology, National City, USA

**Keywords:** alopecia, areata, autoimmune, hair, loss, lupus, erythematosus, systemic

## Abstract

Alopecia areata is an inflammatory, non-scarring hair loss associated with autoimmune conditions. It is more commonly seen with thyroid disorders and vitiligo, but alopecia areata has also been linked to diabetes, psoriasis, rheumatoid arthritis, and systemic lupus erythematosus. Indeed, individuals with alopecia areata have an increased risk of developing systemic lupus erythematosus. A 36-year-old woman presented with hair loss characteristic of alopecia areata. After intralesional injections with triamcinolone acetonide, the areas of hair loss exhibited near complete hair regrowth. Laboratory examination and additional history were suggestive of systemic lupus erythematosus. She was referred to a rheumatologist who confirmed the diagnosis. Awareness of the comorbidities associated with alopecia areata can uncover other autoimmune conditions, such as thyroid disorders and systemic lupus erythematosus. The diagnosis of a new-onset alopecia areata may prompt a deeper investigation of potentially associated conditions.

## Introduction

Systemic lupus erythematosus is a chronic autoimmune disease that can present with anemia, glomerulonephritis, joint pain, oral ulcers, photosensitivity, and seizures. Since 1982, the criteria for the diagnosis of systemic lupus erythematosus have undergone three revisions [1]. The most recent criteria were set in 2019 by the American College of Rheumatology and the European League against Rheumatism. Non-scarring alopecia has been associated with systemic lupus erythematosus and added to the diagnostic criteria as of 2012 [1].

Alopecia areata is an inflammatory, non-scarring hair loss that presents in well-demarcated regions commonly on the scalp. It has been observed with conditions such as diabetes, psoriasis, thyroid disease, and vitiligo [2]. Alopecia areata has also been considered an autoimmune disease similar to rheumatoid arthritis and systemic lupus erythematosus and can be associated with these conditions.

A 36-year-old woman presented with an eight-month history of hair loss for evaluation and treatment. Following the diagnosis of alopecia areata, laboratory studies and additional history established the concurrent diagnosis of systemic lupus erythematosus. The occurrence of alopecia areata in association with systemic lupus erythematosus is discussed.

## Case presentation

A 36-year-old woman presented with an eight-month history of asymptomatic, patchy hair loss. Her history revealed gestational diabetes and iron-deficiency anemia for which she was taking iron supplements.

**Figure 1:**
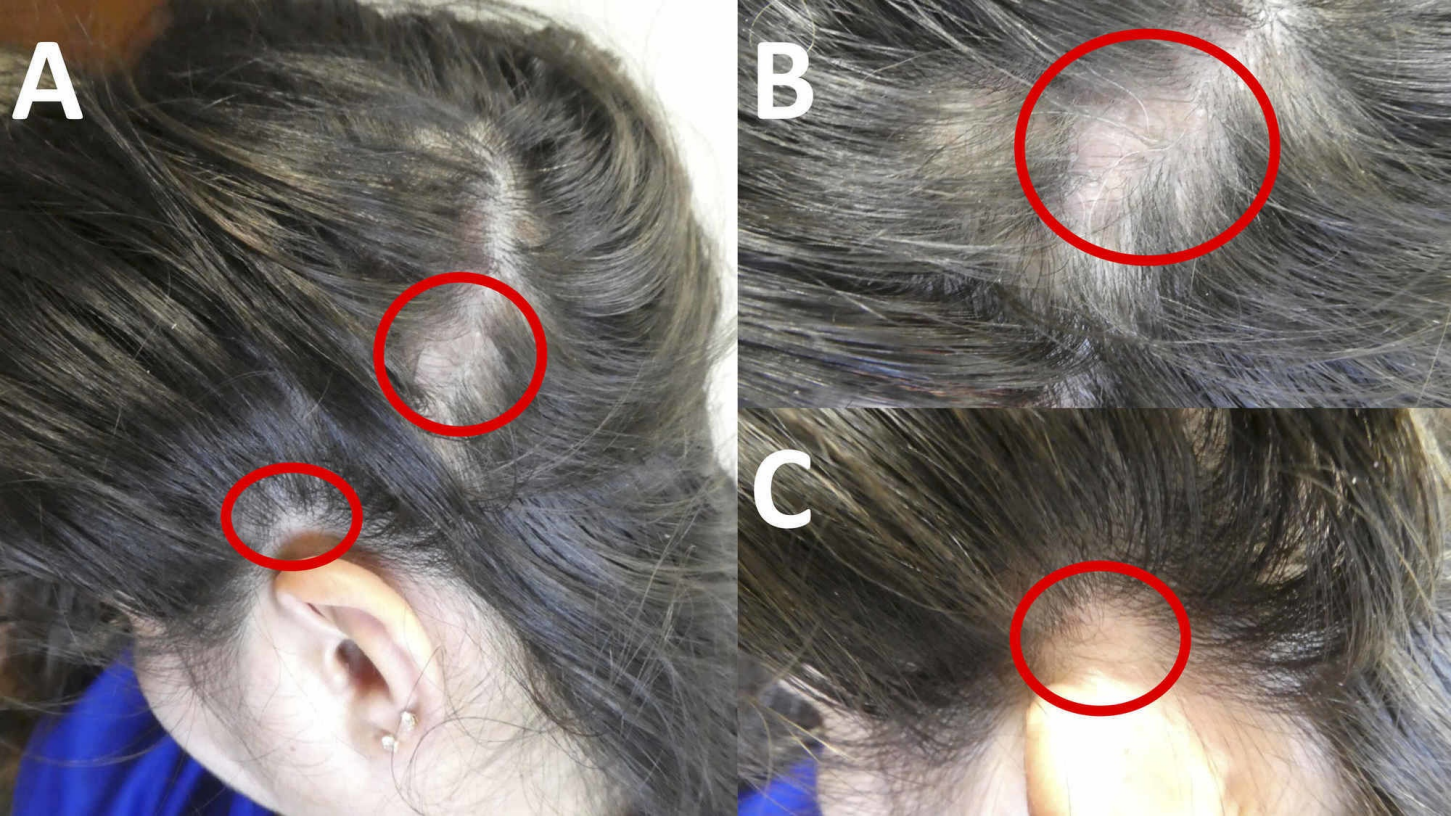
Cutaneous presentation of alopecia areata of the left temporal scalp Distant (A) and closer (B and C) views of a 36-year-old woman’s non-scarring alopecia areata (circled in red) affecting her left temporal scalp: posteriorly (A and B) and above her ear (A and C).

**Figure 2:**
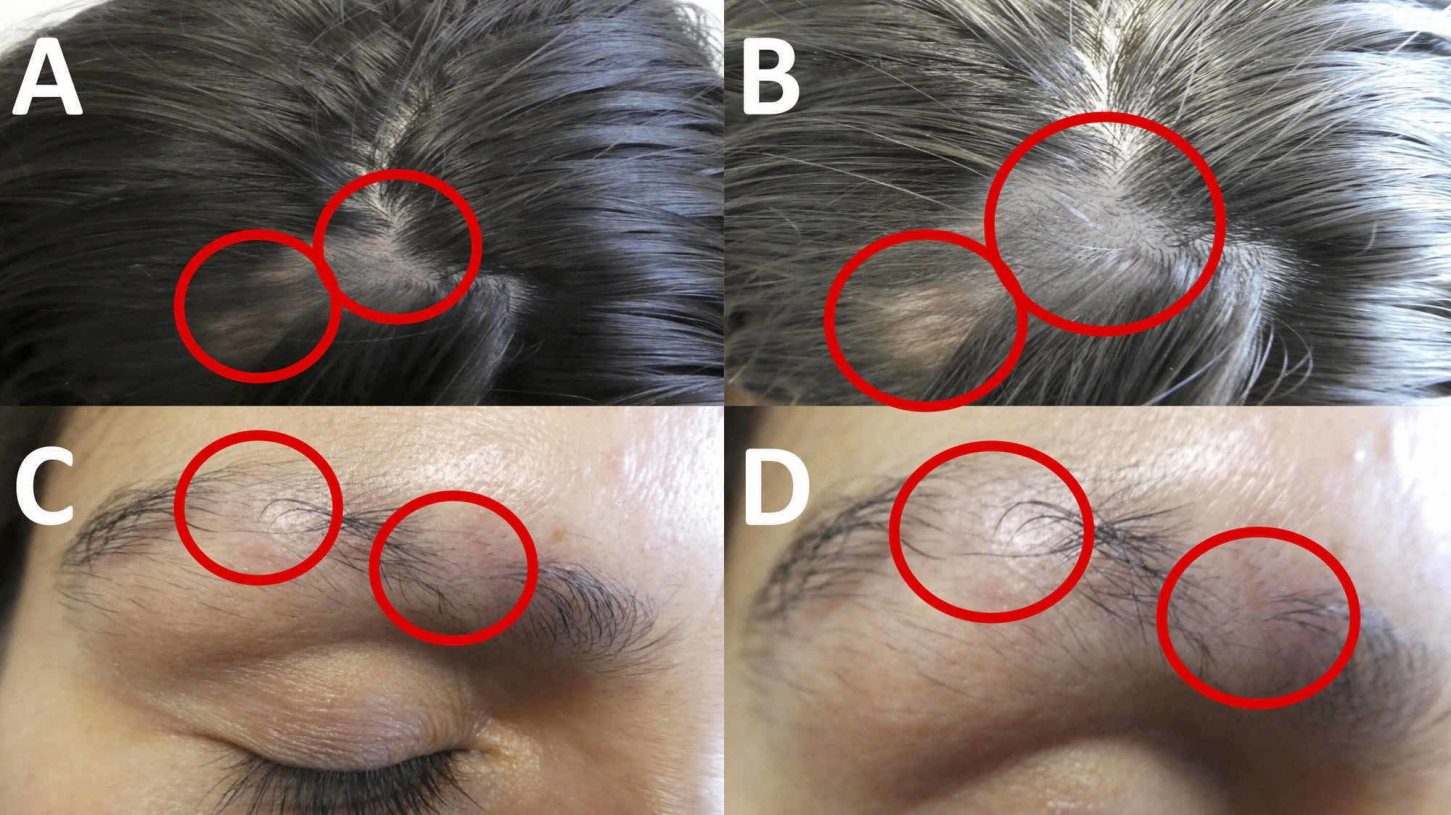
Cutaneous presentation of alopecia areata of the frontal scalp and right eyebrow Distant (A and C) and closer (B and D) views of alopecia areata-related hair loss (circled in red) of the frontal scalp (A and B) and right eyebrow (C and D).

Cutaneous examination, as per patient request, was restricted to above the neck. Annular patches of non-scarring hair loss of the left temporal scalp (posteriorly and above the ear), frontal scalp, and right eyebrow were observed (Figures 1, 2). A biopsy of the affected area of hair loss was declined; therefore, a diagnosis of alopecia areata was established based on the cutaneous evaluation.

Intralesional injections, 2.8 mL of triamcinolone acetonide (3 mg/mL), were administered into both sites of hair loss on the scalp and on the right eyebrow. Near complete regrowth of hair was observed after two treatments of corticosteroid injections that were separated by one month. Her hair loss has not recurred after 12 months of follow up.

Alopecia areata has been associated with other diseases; therefore, laboratory evaluation was performed. Double-stranded DNA (dsDNA) antibody, fasting blood sugar (glucose), rheumatoid factor, SCL70 antibody, Sjogren’s syndrome A (SSA, also referred to as Ro) antibody, Sjogren’s syndrome B (SSB, also referred to as La) antibody, thyroid antibodies (microsomal antibody, peroxidase antibody, and thyroglobulin antibody), and thyroid function tests [thyroid stimulating hormone (TSH), triiodothyronine (T3), and thyroxine (T4)] were in the normal range or negative. However, she was found to have a 1:160 titer of antinuclear antibodies (ANA) with a nuclear dot pattern (normal, less than 1:40), 5.5 antibody index Smith/ribonucleoprotein (Sm/RNP) antibodies (normal, less than 1.0 antibody index), and 29 mg/dL dipstick proteinuria (normal, 5-24 mg/dL). Additional history revealed that she also had photosensitivity and joint pain of her hands and shoulders.

Correlation of the history, clinical features, and laboratory findings were suggestive of systemic lupus erythematosus. The patient was referred to a rheumatologist who confirmed the diagnosis of systemic lupus erythematosus. The patient is planning to begin treatment with hydroxychloroquine.

## Discussion

Alopecia areata is an inflammatory and non-scarring condition. It presents as well-demarcated oval patches of hair loss without erythema or scaling [3]. It affects 1% to 2% of the general population [4].

This autoimmune alopecia is usually located on the scalp and areas of the face, such as the eyebrows, similar to our patient. In men, alopecia areata can also affect the beard [5]. In addition, nail pitting, nail thinning, onychorrhexis, and trachyonychia may be associated with alopecia areata in up to 66% of patients [3].

Hairs at the periphery of the hairless patches are characterized by thin bases and thick apexes and are often referred to as “exclamation point hairs” [3]. In addition, yellow dots of keratin and sebum as well as black dots of broken hairs can help in the visual diagnosis of alopecia areata. More extensive disease involvement can appear with complete loss of hair on the scalp (alopecia totalis) or complete hair loss involving the entire body (alopecia universalis). Less commonly, hair loss can be focused above the ears and involves the lower portion of the frontal, parietal, and temporal regions in a circumferential manner (ophiasis) [3].

Microscopic examination of a skin biopsy of alopecia areata reveals aggregates of lymphocytes around the bulb of the hair shaft [3]. The peribulbar lymphocytes have been likened to a “swarm of bees” in pathology due to the swirling basophilic concentration around the hair shaft [3]. This aggregation of lymphocytes leads to a telogenic shift of hairs and weakening of the hair shaft, producing the characteristic “exclamation point hairs” [2].

Our patient had alopecia areata-related hair loss. In contrast to systemic lupus erythematosus-associated alopecia, there is no fibrosis of the hair follicles observed in alopecia areata. In addition, the basement membrane surrounding the hair shaft and the adjacent epidermis do not show vacuolization or atrophy in alopecia areata. There is no lymphocytic infiltrate along the dermal-epidermal junction or hyperkeratotic follicular plugging in alopecia areata [6].

Comorbid diseases have been observed in patients with alopecia areata. Epigenetic mechanisms (such as DNA methylation and histone modification) and a high soy diet may also be associated with alopecia areata [2]. In addition, thyroid cancer and iron-deficiency anemia are more common among those with alopecia areata [7]. Furthermore, anxiety, depression, and other psychiatric conditions are prevalent among patients with alopecia areata which may be due to the inflammatory effects of stress hormones [2].

Alopecia areata has also been associated with autoimmune diseases. Some of these include diabetes mellitus, psoriasis, rheumatoid arthritis, thyroid disorders, and vitiligo [2].

Several studies have also confirmed the association of alopecia areata with systemic lupus erythematosus [2,4,7,8]. Patients with alopecia areata have a fivefold greater risk of developing systemic lupus erythematosus compared to individuals without alopecia areata [7]. This association is also more common in women, individuals of Jewish ethnicity, and individuals over the age of 60 years [8]. Therefore, in patients with new-onset alopecia areata, it is reasonable to consider laboratory evaluation for other autoimmune diseases, such as ANA (with or without other antibody tests: dsDNA antibody, RNP antibody, SCL70 antibody, Smith antibody, SSA/Ro antibody, and SSB/La antibody), fasting blood sugar (glucose), percent hemoglobin A1c, rheumatoid factor level, and thyroid function test such as TSH, T3, and T4 (with or without additional thyroid antibody tests: microsomal antibody, peroxidase antibody, and thyroglobulin antibody).

The criteria for the diagnosis of systemic lupus erythematosus were recently revised in 2019 by the European League against Rheumatism and the American College of Rheumatology. Both the prior criteria set in 2012 and the current criteria include non-scarring alopecia as a finding of systemic lupus erythematosus. Affected individuals may present with hair loss or visibly fragile and thin hair with broken hairs or both [1].

Interestingly, hair loss is a common finding in the majority of individuals with systemic lupus erythematosus [6]. Patterns of alopecia observed in systemic lupus erythematosus usually include the lupus-specific alopecias associated with either acute lupus erythematosus, discoid lupus erythematosus, subacute cutaneous lupus erythematosus, or tumid lupus erythematosus [6]. However, in a study of 39 patients with systemic lupus erythematosus, 10% developed alopecia areata [9].

The lupus-specific forms of alopecia often exhibit an erythematous or violaceous tone. Some of the alopecia variants result in scarring and an irregular skin pigmentation. In contrast, lupus non-specific alopecias are usually non-scarring and non-erythematous; they include alopecia areata, anagen effluvium, and telogen effluvium [6].

Our patient presented with alopecia areata not only of her scalp but also of her eyebrow. She experienced an excellent clinical response to her alopecia areata treatment. Nearly all of the hair she had lost regrew after two intralesional injections of triamcinolone acetonide, a standard initial approach for the management of patchy alopecia areata [3]. Laboratory studies, based on her diagnosis of alopecia areata, demonstrated positive ANA and Sm/RNP antibodies and proteinuria. These results, accompanied by additional history of photosensitivity and joint symptoms, led to the diagnosis of systemic lupus erythematosus.

## Conclusions

Alopecia areata and systemic lupus erythematosus are both autoimmune diseases. Comorbid conditions associated with alopecia areata include diabetes mellitus, rheumatoid arthritis, systemic lupus erythematosus, and thyroid disorders. An increased risk of developing systemic lupus erythematosus has been observed in individuals with alopecia areata. Our patient presented with alopecia areata which prompted further evaluation and uncovered concurrent systemic lupus erythematosus. Laboratory evaluation for other autoimmune diseases should be considered in patients with new onset alopecia areata.
